# Microbiota of the Digestive Gland of Red Abalone (*Haliotis rufescens*) Is Affected by Withering Syndrome

**DOI:** 10.3390/microorganisms8091411

**Published:** 2020-09-13

**Authors:** Alejandro Villasante, Natalia Catalán, Rodrigo Rojas, Karin B. Lohrmann, Jaime Romero

**Affiliations:** 1 Laboratorio de Biotecnología de los Alimentos, Unidad de Alimentos, Instituto de Nutrición y Tecnología de los Alimentos (INTA), Universidad de Chile, El Líbano 5524, Macul, Santiago 783090, Chile; alejandro.villasante@gmail.com (A.V.); nataliabcatalant@gmail.com (N.C.); 2 Doctorado en Acuicultura, Programa Cooperativo Universidad de Chile, Universidad Católica del Norte, Pontificia Universidad Católica de Valparaíso, Santiago 783090, Chile; 3 Laboratorio de Patobiología Acuática, Departamento de Acuicultura, Facultad de Ciencias del Mar, Universidad Católica del Norte, Larrondo 1281, Coquimbo 1780000, Chile; rrojas@ucn.cl; 4 Centro de Innovación Acuícola AquaPacífico, Larrondo 1281, Coquimbo 1780000, Chile; klohrman@ucn.cl; 5 Departamento de Biología Marina, Facultad de Ciencias del Mar, Universidad Católica del Norte, Larrondo 1281, Coquimbo 1780000, Chile

**Keywords:** withering syndrome, next-generation sequencing (NGS), microbiota, microbiome, abalone, *Haliotis*, *rickettsia*-like organism (RLO), *Candidatus* Xenohaliotis californiensis

## Abstract

Withering syndrome (WS), an infectious disease caused by intracellular bacteria *Candidatus* Xenohaliotis californiensis, has provoked significant economic losses in abalone aquaculture. The pathogen infects gastroenteric epithelia, including digestive gland, disrupting the digestive system and causing a progressive wilting in abalone. Nonetheless, our knowledge about WS implications in digestive gland microbiota, and its role in diseases progress remains largely unknown. This study aims to determine whether digestive gland-associated microbiota differs between healthy red abalone (*Haliotis rufescens*) and red abalone affected with WS. Using high-throughput sequencing of the V4 region of the 16S rRNA gene, our results revealed differences in microbiota between groups. Bacterial genera, including *Mycoplasma*, *Lactobacillus*, *Cocleimonas* and *Tateyamaria* were significantly more abundant in healthy abalones, whilst *Candidatus* Xenohaliotis californiensis and *Marinomonas* were more abundant in WS-affected abalones. Whilst *Mycoplasma* was the dominant genus in the healthy group, *Candidatus* Xenohaliotis californiensis was dominant in the WS group. However, *Candidatus* Xenohaliotis californiensis was present in two healthy specimens, and thus the *Mycoplasma*/*Candidatus* Xenohaliotis californiensis ratio appears to be more determinant in specimens affected with WS. Further research to elucidate the role of digestive gland microbiota ecology in WS pathogenesis is required.

## 1. Introduction

In Chile, aquaculture has become one of the larger food productive sectors, with a sustained expansion rate that has positioned the country in the top ten major aquaculture producers worldwide [1]. However, the Chilean aquaculture industry is highly dependent on salmon culture, which constitutes a risk factor when considering the impact of salmonids disease outbreaks as well as fluctuations in the economy and consumers market. Promoting diversification of Chilean aquaculture is a feasible strategy to ensure the well-being of this industry. Aquaculture of abalone, a marine herbivorous gastropod, including red abalone and Japanese abalone (*Haliotis rufescens* and *Haliotis discus hannai*, respectively), is a promising avenue to achieve this goal, based on a confluence of factors, such as adequate seawater temperature and availability of protected coastal areas for abalone culture, competitive labor cost and an abundant supply of kelp as abalone food [2]. Most of Chilean abalone aquaculture involves intensive farming of red abalone due to facile husbandry management during its life cycle [3]. However, infectious diseases might jeopardize the success of abalone farming in Chile. Withering syndrome (WS) is an infectious disease that was introduced to Chile by importing infected red abalone [4]. The etiology is a *rickettsia*-like organism identified as the gastrointestinal intracellular prokaryote *Candidatus* Xenohaliotis californiensis [5]. WS is characterized by a chronic degenerative condition that causes important economic loss, particularly a severely shrunken body, in abalone aquaculture. *Candidatus* Xenohaliotis californiensis infects gastrointestinal epithelia provoking severe morphological abnormalities within the digestive gland of abalone, which ends in physiological starvation followed by anorexia, cachexia (i.e., absorption of pedal musculature), lethargy and death [5]. However, whether digestive-tract-associated microbiota is modulated in abalone with WS has not been reported to date. It is well known that the gastrointestinal tract harbors an enormous number of microbes within the lumen, which constitute the gut microbiota, an ecosystem in dynamic equilibrium [6]. Gut microbiota carry out essential functions, such as breakdown of foods, uptake of nutrients, enhancement of intestinal development, development and stimulation of mucosal immune system, and antagonistic effects against pathogens, all of which are of relevance to the well-being of the host [6,7]. Host immune system can be modulated by gut microbiota via multiple factors, which include microbial components and their metabolites [7]. Indeed, some infectious diseases have their origin in the disruption of the established community structure and subsequent function changing the overall balance between the microbiota and host, resulting in altered infection susceptibility [8]. Taking this antecedent into account, it is of interest to determine whether WS affects the composition of digestive tract microbiota in abalone, since this is a first step to elucidate how does microbial community respond to WS. Thus, the aim of this study was to characterize the composition of microbiota associated with digestive gland in red abalone with WS. Knowing this aspect of the disease can contribute to a better understanding of the pathogenesis of WS as well as help to implement strategies, including the use of probiotics and/or prebiotics to improve growth performance, inhibition of adherence and colonization of pathogenic bacteria, immune response and WS resistance in red abalone farming.

## 2. Materials and Methods

### 2.1. Sample Collection

Red abalone ranging in size (defined as the maximum length of the elliptical shell) from 35 to 90 mm, were collected from an abalone farm center located at Región de Coquimbo, Chile. During farming, specimens were fed a commercial diet ABLKELP^®^ (specifications at https://www.kelproducts.com/aquacultural). Distinction between healthy specimens (*n* = 5) and specimens with WS disease (*n* = 5) was purely based on the presence of morphological and histological signs. This is because the presence of the pathogens per se is not a feasible criterion for grouping red abalones in healthy or with WS disease, as previously suggested [9]. Moreover Friedman et al. [10] found both low and non-significant Spearman rank correlation coefficients between intensity of *rickettsia*-like organism in abalones with WS and morphological and histological signs (Figure S1). After collection, specimens were placed in sterile bags and preserved on ice until sent to laboratory, where they were euthanized by freezing. All samples were processed within 24 h of collection at Laboratorio de Biotecnología de los Alimentos at Instituto de Nutrición de los Alimentos (INTA) of Universidad de Chile for further analysis. Tissue samples from digestive gland were dissected from specimens, flash frozen in liquid nitrogen and further stored at −80 °C until DNA extraction to perform microbiota analysis.

### 2.2. DNA Extraction and Sequencing

DNA was extracted from digestive gland lysed homogenates using QIAamp PowerFecal DNA Kit (Qiagen, Germantown, MD, USA) according to manufacturer’s protocol. The V4 region of the 16S rRNA gene was amplified following the fusion primer method using primers 515F and 806R as described [11]. The resulting amplicons were of suitable length to be used in the Illumina^®^ Inc. (Shirley, NY, USA) sequencing platform. All PCR reactions were performed in duplicates in a 30-µL reaction mixture containing 1.5 U (5 U/µL) GoTaq^®^ G2 Flexi DNA Polymerase (Promega, Madison, WI, USA), 6 µL of Buffer (5×), 2.4 µL of Mg (25 mM), 1.2 µL of nucleotide mix (5 mM each), 0.3 µL of primers (20 µM) and 18.5 µL of nuclease-free water. In addition, a negative PCR control without DNA template was run. PCR conditions included an initial denaturation at 94 °C for 5 min, followed by 35 cycles of denaturation at 94 °C for 30 s, annealing at 56 °C for 30 s, and extension at 68 °C for 45 s. After the procedure, amplicons from each sample were pooled and run on a 1% agarose gel. Amplicon concentrations were determined using Qubit^®^dsDNA HS Assay kit (Life Technologies, Grand Island, NY, USA). Subsequently, amplicons were purified with QIAquick^®^PCR Purification kit (Qiagen, Germantown, MD, USA). Libraries were sequenced on the paired-end Illumina platform Hiseq PE250 adapted for 300-bp paired-end reads at CD Genomics (http://www.cd-genomics.com).

### 2.3. Bioinformatic Analysis

Quality-filtered reads were assembled into error-corrected amplicon sequence variants (ASVs) using Devisive Amplicon Denoising Algorithm (DADA2) v1.6.0 microbiome pipeline (available at https://github.com/benjjneb/dada2) to identify the presence and abundance of different microbial taxa based on the assembly of the 16S rRNA sequence reads. Forward and reverse reads were truncated at 285 and 275 bp, respectively, by using read quality scores for dataset via filterAndTrim function set with standard parameters (maxN = 0, truncQ = 2, and maxEE = 2). Singleton sequences were automatically removed by DADA2’s error model, followed by a sample inference step using the inferred error model. In addition, chimeric sequences were removed using removeBimeraDenovo function. Assembled ASVs were assigned into the corresponding taxonomy (phylum to genus) level using Silva Database (Silva) naïve Bayesian classifier (implemented in DADA2) and “Silva nr v132 train set” [12]. Using R package Phyloseq [13], we eliminated any ASV without a bacterial phylum assignment, and also those assigned to Cyanobacteria/Chloroplast. Using DADA2, no rarefying of sequence reads was necessary. Finally, Illumina next-generation DNA sequences were deposited in the Sequencing Read Archive (SRA) of the National Centre for Biotechnology Information under Bioproject accession PRJNA636409, SRA run accessions SRX8460649-SRX8460658.

### 2.4. Ethical Notes

The study was conducted in accordance with the guidelines of the Bioethics and Biosecurity Committee of Instituto de Nutrición and Tecnología de los Alimentos (INTA) at Universidad de Chile.

### 2.5. Statistical Analysis

Statistical analyses were performed using “R” v. 3.4.3 (http://www.R-project.org). R Packages Phyloseq and Vegan [14] were used for microbiota data analyses. Alpha diversity measured by Shannon and Simpson diversity index and species richness measured by Chao1 was calculated using Phyloseq. Normality was tested with Shapiro–Wilk test and alpha diversity indexes were further analyzed for differences using a Student’s *t*-test with a 5% of significance level. Unweighted and Weighted UniFrac distances were calculated as β diversity measures using Phyloseq package in R. To statistically test the homogeneity of microbial community composition, we performed permutational multivariate analysis of variance (PERMANOVA) using package Vegan analyses on distance metrics. Differential taxa abundance was performed using LefSe [15]. This method involves Kruskal–Wallis (KW) sum-rank test between classes of data followed by (unpaired) Wilcoxon rank-sum test to conduct pairwise tests among subclasses. LDA is then used to estimate the effect size for each of the identified taxa. We used LEfSe (Galaxy Version 1.0) with default parameters (KW = 0.05; Wilcoxon = 0.05; LDA score threshold = 2.0) as well as using the all-against-all strategy for multi-class analysis. All other comparisons were made using either Welch’s *t*-test or Kruskal–Wallis (KW). Mann–Whitney U test was conducted to detect differences in *Mycoplasma*/*Candidatus* Xenohaliotis californiensis ratio between groups.

## 3. Results

### 3.1. High-Throughput Sequence Data

A total of 1,018,662 initial raw reads were obtained, from which 646,093 reads were retained after removing low-quality reads and chimeras (Table S1). Further, 306,616 reads were measured in the group of healthy red abalones and 339,477 reads were measured in the group of red abalones with WS.

### 3.2. Analysis of Microbiota Diversity

**Figure 1:**
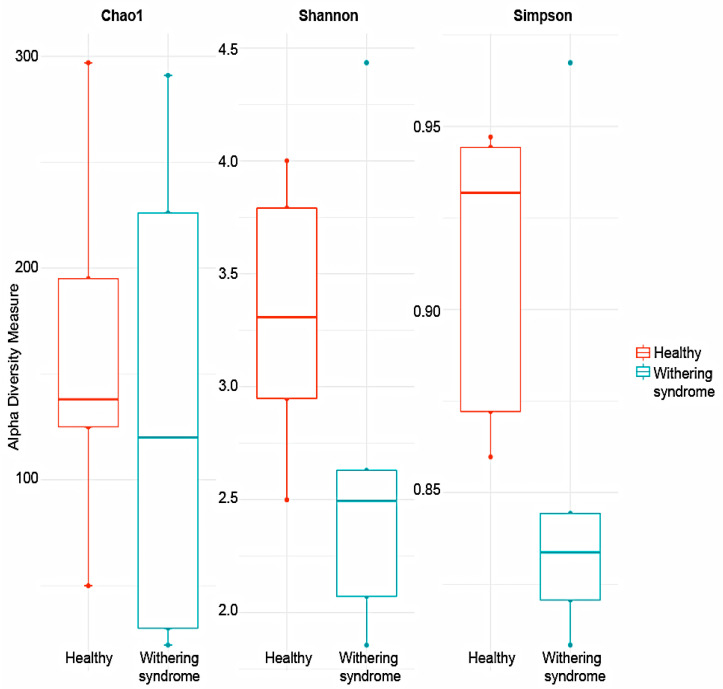
Comparison of alpha diversity indexes; Chao 1, Shannon and Simpson, between healthy red abalones and red abalones affected with withering syndrome disease. Note the different scale in Y-axis due to different indexes ranging values.

**Figure 2:**
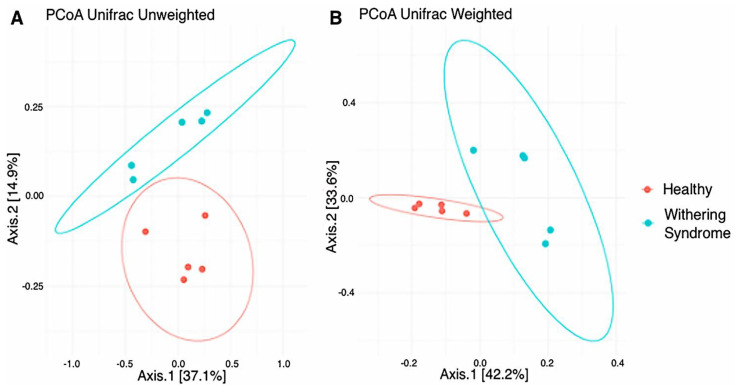
Principal coordinates analysis (PCoA) of the bacterial communities derived from the unweighted (**A**) and weigthed (**B**) UniFrac distance matrix. Circles represent individual samples from red abalone digestive gland microbiota. Red circles correspond to samples from healthy abalones (*n* = 5) and blue light circles correspond to samples from red abalones affected with withering syndrome disease (*n* = 5).

No significant differences in richness (Chao1: *p* < 0.739) and either alpha diversity index (Shannon: *p* < 0.283; Simpson: *p* < 0.140) between healthy red abalones and red abalones with WS were observed after *t*-test (Figure 1). Regarding beta diversity, the composition of digestive gland microbiota was significantly (*p* = 0.009) different between healthy red abalones and red abalones with WS when considering weighted distances (quantitative UniFrac phylogenetic distance matrices). However, no differences (*p* = 0.177) between groups were detected when considering unweighted distances (qualitative UniFrac phylogenetic distance matrices). In order to depict similarity in digestive gland microbiota obtained from both healthy red abalones and red abalones with WS (based on unweighted and weighted UniFrac analyses) the principal coordinates plot (PCoA) was used (Figure 2A and B, respectively). In the PCoA of the unweighted UniFrac analyses, the two main components explained 52% of the total variance (axis 1, 37.1%; axis 2, 14.9%) while in the PCoA of the weighted UniFrac analyses, the two main components explained 75.8% of the total variance (axis 1, 42.2%; axis 2, 33.6%).

### 3.3. Taxonomic Composition and Differential Abundance of Bacterial Communities of Digestive Gland of Healthy Red Abalone and Red Abalone with WS Disease

**Figure 3:**
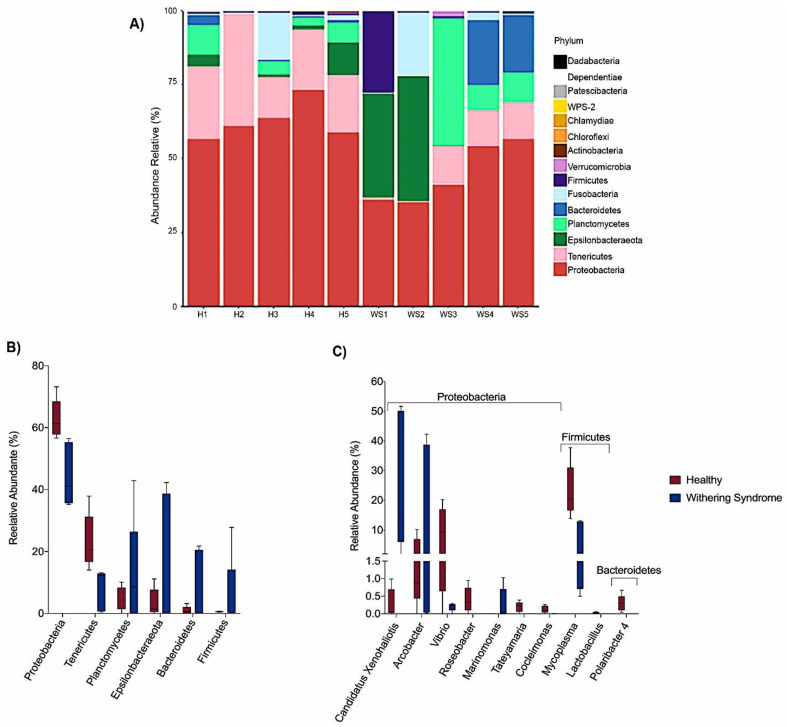
(**A**) Relative abundance (%) at phylum level for each sample in digestive gland microbiota from healthy red abalone (H1, H2, H3, H4 and H5) and red abalone affected with withering syndrome disease (WS1, WS2, WS3, WS4 and WS5). Comparison of digestive gland microbiota from healthy red abalone (red boxes) and red abalone affected with Withering Syndrome (blue boxes) in terms of relative abundance (%) at phylum level (**B**) and genus level (**C**).

**Figure 4:**
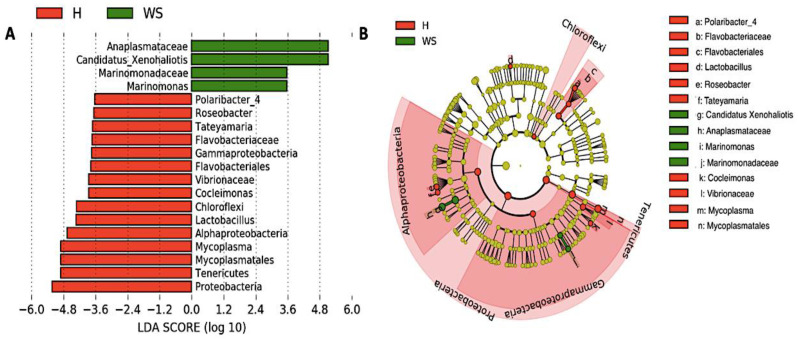
Differences in digestive gland microbiota of healthy red abalones (H) compared with red abalones with withering syndrome disease (WS). Analysis of 16S rRNA reveals the differential composition of microbiota depending on the origin of the sample (H or WS). LEfSe was used to determine the statistical significance and the effect size of the differential abundance of taxa between H and WS. Section (**A**) shows LDA score of abundance of taxa; Section (**B**) shows cladogram showing differentially abundant taxa (phylum to genus) of digestive gland microbiota in healthy red abalones and red abalones with WS disease.

Taxonomic composition reveled *Proteobacteria*, *Tenericutes* and *Planctomycetes* were the dominant phyla in digestive gland microbiota from healthy red abalones (62.8%, 23.4% and 4.8%, respectively) while *Proteobacteria*, *Epsilonbacteraeota* and *Planctomycetes* were the most abundant phyla in red abalones with WS (44.6%, 15.5% and 12.3%, respectively; Figure 3A,B). At the genus level, the dominant bacteria genus in digestive gland microbiota from healthy red abalones were *Mycoplasma* (23.1%), *Vibrio* (8.9%) and *Arcobacter* (3.1%) (Figure 3C). *Candidatus* Xenohaliotis californiensis and *Arcobacter* were the dominant bacteria genus detected in the digestive gland microbiota in red abalones with WS (25.1% and 15.5%, respectively; Figure 3C). Further, significant differences in relative abundance (LDA effect side score ≥ 3.5; Figure 4A) at different taxon levels (using phylum to genus-level data) between healthy red abalones and red abalones with WS were detected by LEfSe analysis. These differences are illustrated in a cladogram in Figure 4B. Phylum *Proteobacteria*, *Tenericutes* and *Chloroflexi* were differentially abundant in healthy red abalones compared with red abalones with WS (Figure 4A). Class *Alphaproteobacteria* and *Gammaproteobacteria* were differentially abundant in healthy red abalones compared with red abalones with WS. Order *Flavobacteriales* and *Mycoplasmatales* were significantly more abundant in healthy red abalones compared with red abalones with WS. At the family level, *Flavobacteriaceae* and *Vibrionaceae* were significantly more abundant in healthy red abalones compared with red abalones affected with WS, and at the bacteria genus level, *Mycoplasma*, *Lactobacillus*, *Cocleimonas*, *Tateyamaria*, *Roseobacter* and *Polaribacter_4* were significantly more abundant in healthy red abalones compared with red abalones with WS. On the other hand, in red abalones with WS, bacterial family *Anaplasmataceae* and *Marinomonadaceae* as well as bacteria genera *Candidatus* Xenohaliotis californiensis and *Marinomonas* were significantly more abundant compared with healthy red abalones. Finally, we observed that the *Mycoplasma*/*Candidatus* Xenohaliotis californiensis ratio was significantly (*p* = 0.011) lower in red abalones affected with WS compared with healthy specimens (0.3 *v/s* 76.8, respectively).

## 4. Discussion

Red abalone are the primary species of abalone aquaculture production in Chile. Withering syndrome is a chronic, slow progressing disease causing important economic loss (i.e., low growth rate, reduced weight condition index and high mortality) in red abalone farming. It has been said that the causative agent is a *rickettsia*-like organism known as *Candidatus* Xenohaliotis californiensis, which initially infects the epithelium of the gastroenteric system (i.e., posterior esophagus and digestive gland) followed by a progressive deterioration in the mussel pedal muscle [5,10]. Although this is an infectious disease that initially affects the digestive tract of abalone, no study on potential changes of microbiota associated with digestive tract (i.e., digestive gland) of abalone with WS has been conducted. Thus, the present study is the first report describing structural changes in the bacterial communities of digestive gland microbiota in red abalones affected with WS.

Taxonomic composition analyses revealed differences in the ranking of major bacteria phyla of digestive gland microbial communities between both conditions in red abalones (healthy specimens: *Proteobacteria* > *Tenericutes* > *Planctomycetes* and specimens with WS disease: *Proteobacteria* > *Epsilonbacteraeota* > *Planctomycetes*). However, in both conditions, *Proteobacteria* was the dominant bacteria phylum in digestive gland microbiota. In agreement with our results, previous works have reported *Proteobacteria* as dominant phylum in digestive tract tissues in several marine gastropods species. Neu et al. [16] reported *Proteobacteria* to be the most abundant phylum in the digestive gland and whole-body samples of intertidal gastropods species, such as owl limpet (*Lottia gigantea*), rough periwinkle (*Littorina keenae*) and black turban snail (*Chlorostoma funebralis*). Ito et al. [17] found that *Proteobacteria* as the most abundant phylum in the gut microbiota of marine gastropods species, including sea hare (*Dolabella auricularia*) and sea snail (*Batillus cornutus*). Moreover, *Proteobacteria* have been reported to be the dominant phylum in the gut microbiota of marine bivalve molluscs, such as blue mussel (*Mytilus edulis*). However, the dominance of *Proteobacteria* phylum in digestive tract-associated microbiota in molluscs is not consistent across the literature. For instance, Gobet et al. [18] detected *Fusobacteria* as the dominant phylum of microbiota associated with the digestive gland in European abalone (*Haliotis tuberculate*) fed different macroalgae-based diets (*Palmaria palmata*, *Ulva lactuca*, *Saccharina latissima*, *Laminaria digitate*). Aceves et al. [19] observed *Tenericutes* was the dominant phylum, followed by *Proteobacteria*, *Fusobacteria* and *Bacteroidetes* in the digestive gland microbiota of the freshwater mussel Alabama rainbows (*Villosa nebulosa*). Therefore factors, including host species, digestive tract section, diet composition, seasonal patterns and environment (i.e., freshwater or seawater, water temperature and biogeography) most likely influence digestive-system-associated microbial communities of the host.

Our results indicate that heathy and diseased abalone had similar microbiota species diversity, in terms of both species richness (Chao Index) and abundance levels (Shannon Index). However, we observed significant differences between healthy and diseased groups in terms of the phylogenetic distances of the microbiota composition when considering the weighted UniFrac analysis, which accounts for the presence and absence of species. We can attribute this variation to *Mycoplasma* species and *Candidatus* Xenohaliotis californiensis; for example, digestive gland microbiota were strongly dominated (23.09%) by genus *Mycoplasma* in healthy red abalones whilst *Mycoplasma’s* share of digestive gland microbiota decreased to 7.88% in red abalones with WS disease (Figure 3B). On the contrary, *Candidatus* Xenohaliotis californiensis, causative agent of WS disease, was dominant (25.12%) genus in digestive gland microbiota of red abalones with WS disease while *Candidatus* Xenohaliotis californiensis’s share of digestive gland microbiota decreased to 0.7% in healthy red abalones. Moreover, these observed differences in relative abundance were significant based on LEfSe analysis, which revealed that order *Mycoplasmatales* and genus *Mycoplasma* were significantly more abundant in healthy red abalones, whilst genus *Candidatus* Xenohaliotis californiensis was significantly more abundant in red abalones with WS disease (Figure 4A). Interestingly, recent research has detected *Mycoplasma* in high abundance in algae [20,21], which suggests that feeding an algae-based diet to abalone might constitute a route of access into digestive system for *Mycoplasma*. Similar to our study, previous works have reported that genus *Mycoplasma* either exhibits a preponderant share of digestive system-associated microbiota or a ubiquitous presence (member of the core microbiota) in digestive microbiota in several mollusc species. For example, Cicala et al. [22] found that the genus *Mycoplasma* dominated gastrointestinal microbiome in northeast Pacific blue (*Haliotis fulgens*) and yellow (*Haliotis corrugate*) abalone. Gobet et al. [18] found that the genus *Mycoplasma,* along with genera *Psychrilyobacter* and *Vibrio,* were part of the core microbiota, constantly dominating the digestive gland microbiota, in the European abalone. In other mollusc species, such as the freshwater mussel Alabama rainbows, digestive gland microbiota was overwhelmingly dominated by genus *Mycoplasma* [19]. The authors suggested that *Mycoplasma*-like sequences could not be ascribed unequivocally to the genus *Mycoplasma* and probably represent new lineages within the class *Mollicutes*. Aronson et al. [23] observed that the genus *Mycoplasma* was predominant in the bacterial community of the digestive gland and stomach of the gastropod deep-sea, bone-eating snail (*Rubyspira osteovora*), while Neu et al. [16] highlighted that the high abundance of genus *Mycoplasma* was characteristic of microbial communities of gastropod species, such as C. *funebralis, C. eiseni* and *L. gigantea.* Similarly, Pierce and Ward [24] reported that the genus *Mycoplasma* was a member of the core bivalve gut microbiome in both eastern oyster and blue mussel. Although *Mycoplasma* has been considered an obligate vertebrate pathogen causing several diseases in humans [25], the consistently observed high abundance of this genus in gastrointestinal-associated microbiota in healthy bivalves and gastropods species suggests that *Mycoplasma* or *Mycoplasma*-like organisms play an important ecological role in the gut and health of various marine molluscs. In line with this statement, Register et al. [26] proposed that *Mycoplasma* might be highly host-specific and Wang et al. [27] suggested the existence of a mutualistic relationship between *Mycoplasma* and their hosts. This idea was further supported by Cicala et al. [22] who suggested that *Mycoplasma* contribute to their abalone host by playing pivotal roles in several metabolic functions. In this regard, mutualism between *Mycoplasma*-like symbionts and their host is built upon *Mycoplasma’s* inability to perform many basic metabolic functions due to their small genomes [28]. An example of this mutualism is that *Mycoplasma* require molecules such as sterols and fatty acids provided by a host to survive [29] while *Mycoplasma* contribute to animals by digesting nutrient-poor foods as well as supplying their hosts with amino sugars and simple carbohydrates. This is especially true, as the *Mycoplasma* genome presents a high number of genes involved in the degradation of glycans, proteins and complex oligosaccharides [27,30,31]. Another benefit of this aforementioned mutualism is that *Mycoplasma* might protect their hosts against microbial pathogen infections by competitive inhibition of the binding site at the epithelium cell surface of the host. *Mycoplasma* strongly adheres to host cell surfaces using outer membrane proteins, such as adhesin [32], thus blocking microbial pathogens binding to the epithelium cell surface of host. In addition, *Mycoplasma* likely protects their hosts against microbial pathogen infections by breaking down sialic acid residues of outer membranes proteins used by microbial pathogens to avoid the host’s innate immune response, based on the presence of sialic acid lyase genes in *Mycoplasma* genome [27,33].

In our study, *Mycoplasma* was the dominant genus in healthy specimens and was sharply reduced in specimens with WS disease, while quite the opposite occurred with genus *Candidatus* Xenohaliotis californiensis. Indeed, we observed the presence of this *Candidatus* Xenohaliotis californiensis in digestive gland microbiota in two healthy specimens, suggesting that the mere presence of this pathogen might not be enough to cause WS disease [9,22], and thus other cofactors might be required to promote the proregression of the disease. Therefore, we hypothesize that WS disease requires the presence of the *Candidatus* Xenohaliotis californiensis along with a significant drop in the relative abundance of *Mycoplasma,* causing a concomitant decrease in *Mycoplasma*/*Candidatus* Xenohaliotis californiensis ratio, as observed in our study. A potential mechanism of *Mycoplasma*-induced protection might be based on the presence of sialic acid lyase activity degrading the α 2,3 sialic acid residues at the receptor domains of that host cell, which are recognized by the outer membrane proteins A (OmpA) of *rickettsia*-like organism [34]. The OmpA has previously been described as putative adhesin favoring *Rickettsia* attachment to epithelial cells in vitro [35]. Further, this receptor–ligand interaction has been referred to as essential for the efficient cellular entry of *Anaplasma phagocytophilum*, a member of the order Rickettsiales, into human myeloid cells [36]. Remarkably, the authors described that competitive inhibition of binding and entry using OmpA can be achieved by either a monoclonal antibody directed against the sLex α 2,3 sialic acid residue of the P-selectin glycoprotein1 (PSGL-1) receptor or sialidase treatment of host cell. 

In addition, the genus *Lactobacillus* was significantly more abundant in healthy specimens in our study. However, *Lactobacillus’s* share of digestive gland microbiota was quite low in healthy red abalones (Figure 3B). *Lactobacillus* is a member of lactic acid bacteria (LAB), which have been used as probiotics in animal production systems and fish aquaculture, due to their role in adjusting intestinal environment, regulating intestinal mucosal immunity and maintaining intestinal barrier function [37,38,39]. In shellfish aquaculture industry most of the reported beneficial effects of these probiotics, including improved growth performance, enzymatic contribution to nutrition, inhibition of adherence and colonization of pathogenic bacteria in digestive tract, modulation of gut microbiota, and increase hematological parameters and immune response, have been observed in shrimp farming [40]. On the other hand, less antecedents exist regarding LAB administration as probiotics in marine mussel’s aquaculture [39,41,42,43,44]. Therefore, contrary to what has been observed in fish and shrimp aquaculture species, our results suggest that *Lactobacillus’s role* adjusting intestinal environment and promoting health in abalone host may be marginal. 

In recent years, there has been growing consumer concern regarding the indiscriminate use of antibiotics to treat infectious diseases in aquaculture farming due to the development and dissemination of antibiotic resistance, food safety hazards and environmental issues, such as residue accumulation and aquatic biodiversity toxicity [40,45]. As a reaction to this concern, in order to ensure safe aquaculture food production, several government agencies have established maximum residual limits for antibiotics in food [45]. In other more extreme cases, such as the European Union, the use of antibiotics in food production has been banned since 2003 [40]. Therefore, efforts should be focused on reducing antibiotics to safeguard the environment and ensure safety of consumers, feed industry and aquaculture workers [45]. In this regard, the use of probiotics is a feasible supplementary strategy to vaccine and chemicals in aquaculture [40]. Based on this fact, it is of interest to explore whether the administration of *Mycoplasma* to promote red abalone health could constitute a strategy to improve productivity in red abalone farming. This is especially true since WS disease management should be approached from a sustainable point of view considering aspects such as whether methods are environmentally friendly, safe for human consumption and cost-effective.

## 5. Conclusions

This study provided evidence of differences in the structure of bacteria communities of red abalone digestive microbiota between healthy specimens and specimens with WS disease. *Mycoplasma* and *Candidatus* Xenohaliotis californiensis were the most abundant genera, and their relative abundance most strongly contributed to the microbial profile variations between diseased and healthy abalone in this study. Similar to previous studies, we observed that the mere presence of *Candidatus* Xenohaliotis californiensis was not associated with WS in abalone; however, a decrease in the *Mycoplasma*/*Candidatus* Xenohaliotis californiensis ratio appears to be more indicative of specimens affected with the disease. Further research to explore the potential use of *Mycoplasma* as a probiotic to promote abalone health, and thus improve productivity in farming, is required.

## Supplementary Materials

The following are available online at https://www.mdpi.com/2076-2607/8/9/1411/s1, Figure S1: Macroscopic and histologic images of healthy and WS-affected abalones. Table S1: Reads obtained from healthy and disease specimens from Illumina sequencing.
