# Supplementary material for: Microbiota of the Digestive Gland of Red Abalone (Haliotis rufescens) Is Affected by Withering Syndrome
Source: Microorganisms. 2020 Sep 13;8(9):1411. doi: 10.3390/microorganisms8091411 (PMC7565822; doi:10.3390/microorganisms8091411)
Supplement: Supplementary file 1 [file microorganisms-08-01411-s001.pdf]

Supplementary material

Figure 1S. Macroscopic and histologic images of healthy and WS-affected abalones.

A B C D E F

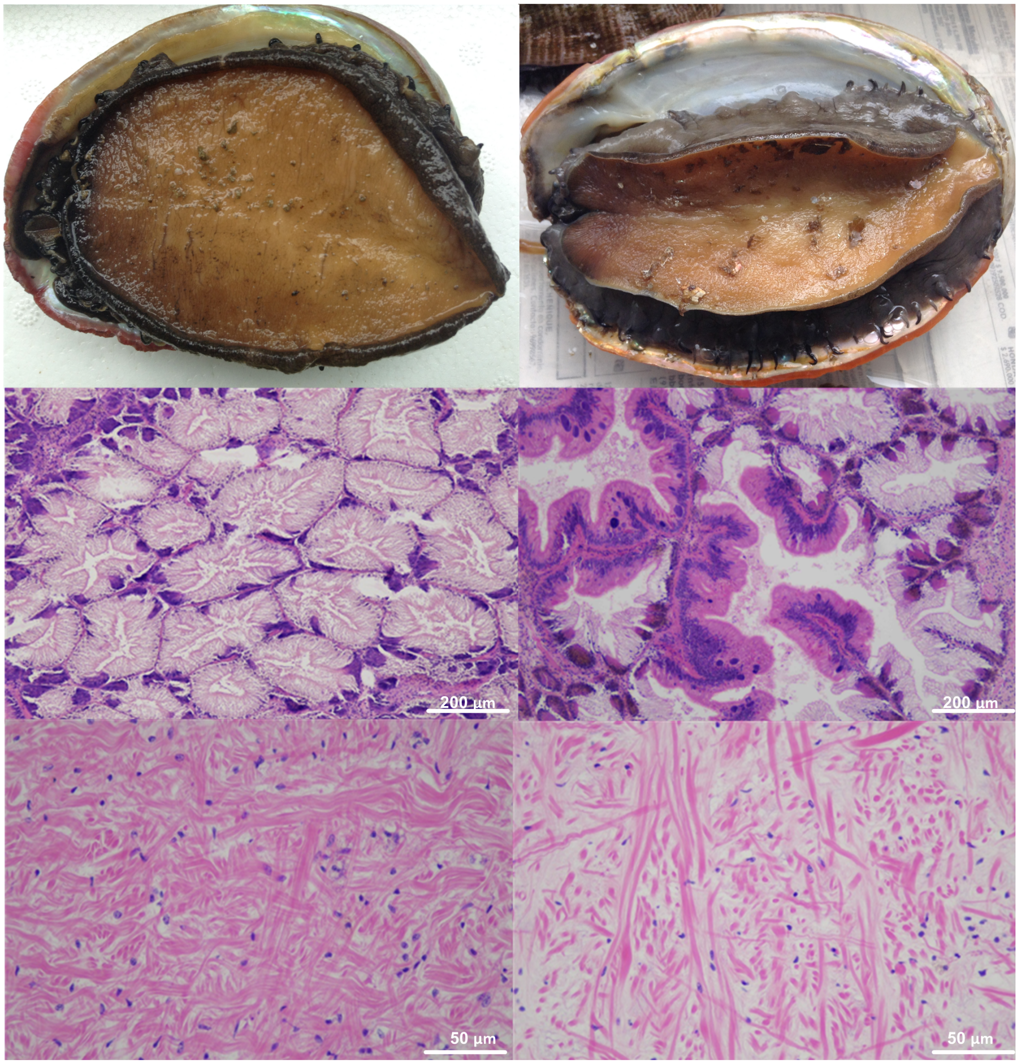

The figure shows three images of an apparently healthy abalone without pathognomonic signs of withering syndrome on the left (figures A, C, E), and the three images on the right (B, D, F), correspond to an infected animal.

A: Macroscopic image of an abalone that looks healthy, no signs of the disease, with a full foot, that fills the whole shell.

B: Macroscopic image of a diseased abalone with a retracted and “withered” foot.

C: Normal and healthy digestive gland, the digestive tubules perform intracellular digestion.

D: End stage of the disease in red abalone: metaplasia (adult tissue turning to another adult tissue, which is irreversible). *Candidatus* Xenohaliotis californiensis, the bacterium that causes withering syndrome, cannot invade the digestive diverticulae, so it apparently triggers the transformation of this tissue to a postesophagous-like tissue, where it thrives. As seen in this picture, there is only a small part of digestive tissue left, so the abalone can not digest its food, and has to digest his foot proteins.

E: healthy abalone foot, with plenty of muscle fibers.

F: Abalone with metaplasia with diminished muscle fibers.

Table S1.- Reads obtained from healthy and disease specimens from Illumina sequencing.

Sample healthy	input	nonchim	Sample WS	input	nonchim
H1	60323	32754	WS1	67794	56938
H2	55290	34750	WS2	65904	40443
H3	60913	34016	WS3	67165	38845
H4	50991	34977	WS4	241500	165430
H5	296989	170119	WS5	51793	37821
Total Healthy	524506	306616	Total WS	494156	339477

Input are raw reads.
Nonchim are reads after filtering chimeras.
